# What differentiates primary care physicians who predominantly prescribe diuretics for treating mild to moderate hypertension from those who do not? A comparative qualitative study

**DOI:** 10.1186/1471-2296-13-9

**Published:** 2012-02-29

**Authors:** Christian M Rochefort, Julia Morlec, Robyn M Tamblyn

**Affiliations:** 1 Clinical & Health Informatics Research Group, Department of Medicine and Department of Epidemiology, Biostatistics and Occupational Health, McGill University & McGill University Health Center, 1140 Pine Avenue West, Montreal, QC, H3A 1A3, Canada

**Keywords:** Hypertension, Antihypertensive treatments, Physician, Prescribing behavior, Cost-effectiveness, Qualitative study

## Abstract

**Background:**

Thiazide diuretics are cost-effective for the treatment of mild to moderate hypertension, but physicians often opt for more expensive treatment options such as angiotensin II receptor blockers or angiotensin converting enzyme inhibitors. With escalating health care costs, there is a need to elucidate the factors influencing physicians' treatment choices for this highly prevalent chronic condition. The purpose of this study was to describe the characteristics of physicians' decision-making process regarding hypertension treatment choices.

**Methods:**

A comparative qualitative study was conducted in 2009 in the Canadian province of Quebec. Overall, 29 primary care physicians--who are also participating in an electronic health record research program--participated in a semi-structured interview about their prescribing decisions. Physicians were categorized into two groups based on their patterns of prescribing antihypertensive drugs: physicians who predominantly prescribe diuretics, and physicians who predominantly prescribe drug classes other than diuretics. Cases of hypertension that were newly started on antihypertensive therapy were purposely selected from each physician's electronic health record database. Chart stimulated recall interview, a technique utilizing patient charts to probe recall and provide context to physician decision-making during clinical encounters, was used to elucidate reasons for treatment choices. Interview transcripts were synthesized using content analysis techniques, and factors influencing physicians' decision making were inductively generated from the data.

**Results:**

We identified three themes that differentiated physicians who predominantly prescribe diuretics from those who predominantly prescribe other drug classes for the initial treatment of mild to moderate hypertension: a) perceptions about the efficacy of diuretics, b) preferred approach to hypertension management and, c) perceptions about hypertension guidelines. Specifically, physicians had differences in beliefs about the efficacy, safety and tolerability of diuretics, the most effective approach for managing mild to moderate hypertension, and in aggressiveness to achieve treatment targets. Marketing strategies employed by the pharmaceutical industry and practice experience appear to contribute to these differences in management approach.

**Conclusions:**

Physicians preferring more expensive treatment options appear to have several misperceptions about the efficacy, safety and tolerability of diuretics. Efforts to increase physicians' prescribing of diuretics may need to be directed at overcoming these misperceptions.

## Background

Hypertension is one of the most prevalent chronic conditions worldwide. The age--and sex-adjusted prevalence of hypertension is estimated to be 28% among North American countries, whereas it is estimated to be as high as 44% in European countries [1-3]. Hypertension has been identified as the most important modifiable risk factor for cardiovascular morbidity and mortality [4,5], and pharmacotherapy is an important aspect of hypertension management [6]. Numerous pharmacological agents exist for the treatment of hypertension, including thiazide diuretics, angiotensin converting enzyme inhibitors (ACEIs), angiotensin II receptor blockers (ARBs), calcium channel blockers (CCBs), and beta-blockers (BBs) [7-9], and these agents vary considerably in cost [7].

While there is evidence that specific pharmacological agents have benefits for patients with particular co-morbidities (e.g., diabetes or nephropathy) [8], for the majority of patients with mild to moderate hypertension, the major randomized control trials [10,11], meta-analyses and systematic reviews [12-16] have provided evidence that initial monotherapy with a thiazide diuretic is as equally effective as the other therapeutic agents in preventing cardiovascular mortality and morbidity. For this reason, and because they are the least costly agents [7], many evidence-based guidelines have recommended that low doses of diuretics should be considered as the first-line treatment of mild to moderate uncomplicated hypertension [9,17,18].

Despite this evidence, studies demonstrate that many physicians favour more expensive alternatives such as angiotensin II receptor blockers (ARBs) or angiotensin converting enzyme inhibitors (ACEIs) for the initial treatment of patients with mild to moderate uncomplicated hypertension [6,19]. This observation is striking given the escalation of health care expenditures, and given that hypertension is one of the most costly conditions treated by primary care physicians [20]. It is also remarkable given that access to affordable medications has been identified as an important determinant of adherence to and persistence with hypertension treatment [19,21,22], and thus of improved health outcomes. To improve the cost-effectiveness of hypertension management and patients' compliance with hypertension treatment, there is a need to have a better understanding of the factors that influence physicians' decision making when selecting an antihypertensive treatment. The purpose of this study was to describe the characteristics of physicians' decision-making process regarding hypertension treatment choices.

## Methods

### Context

The study was conducted in Quebec, Canada, a province with 8.5 million residents and 16, 000 physicians. The provincial insurance agency (RAMQ) provides health insurance for all provincial residents, and pays all physicians and community pharmacists on a fee-for-service basis. Beneficiary, medical billing and pharmacy claims data can be used to create longitudinal health histories for each patient. This data has been validated [23] and is frequently used for health services and epidemiological research [24,25].

Since 2003, the Medical Office of the 21st Century (MOXXI) [26,27], an experimental community-based clinical information system (CIS), links these databases and integrates this information into an electronic health record system to support clinical decision-making. This electronic health record provides physicians with: (1) a daily updated drug profile for each of their patients that reflects current and past medications as well as drug costs; (2) a list of health problems that includes diagnoses recorded on billings by all physicians who treat a given patient; (3) dates and reasons for emergency room visits and hospitalizations. In addition, the MOXXI system has additional features to support clinical management and decision making, including an electronic prescribing system that records treatment indications and adds them to the problem list as well as reasons for drug discontinuation orders (documentation of adverse drug events), information on past treatment, drug/allergy and disease alerts, and various experimental decision support systems. Taking advantage of this CIS, we sought to describe the characteristics of physicians' decision-making process regarding hypertension treatment choices.

### Study design & participants

A comparative qualitative study, using chart stimulated recall interview [28-30], was conducted to elucidate differences in clinical policies that would lead physicians to prescribe cost-effective first line treatment (thiazide diuretics) rather than alternate therapeutic classes or multiple therapies to patients with mild to moderate uncomplicated hypertension within their practice. Chart stimulated recall interview is a technique by which a physician uses documentation of actual patient encounters to stimulate recall of his or her own decision-making processes while an evaluator probes the reasoning behind the decision-making [28,30]. Chart stimulated recall interviews help address the well described discrepancy between physicians' perceived and actual behaviors, as well as recording bias inherent in methods based on chart review [28]. Several studies support the validity of this method for assessing the cognitive decision-making processes that influence performance [28-30].

Overall, 29 of the 80 primary care physicians using the MOXXI clinical information system (CIS) agreed to participate in a chart stimulated recall analysis of their prescribing decisions. The major reasons for declining to participate in the study were lack of time or interest. For the participating physicians, we used the MOXXI CIS system to purposely sample patients from each physician's practice who represented variations in a given physician's approach to hypertension management. We restricted the analyses to patients who were newly started on therapy (incident patients) so that we would analyse only those patients for whom the study physician was making the initial therapeutic decision. This restriction was necessary because primary care physicians often renew medications started by other physicians and their prescribing practices in these instances do not necessarily reflect their own clinical decisions [31]. We identified incident hypertension patients as those who had a diagnosis of hypertension by the study physician and no prior prescription for an antihypertensive drug. We first stratified all incident patients started on therapy within the last year into two groups: (1) started on diuretics alone or (2) started on calcium channel blockers (CCBs), angiotensin II receptor blockers (ARBs), angiotensin converting enzyme inhibitors (ACEIs), beta-blockers (BBs) either alone or in combination with other drugs. We then stratified physicians into two groups: 1) prescribed diuretics as first-line therapy for most patients, or 2) never or rarely prescribed diuretics as first-line therapy. For each physician, we randomly sampled two incident patients for the chart stimulated recall interview; one on which the physician prescribed diuretics as first line treatment and a second where alternate therapy was selected. For physicians who never or rarely prescribed diuretics, we randomly selected two patients who were started on alternate treatment.

### Chart stimulated recall

A semi-structured interview guide was developed from the literature to elucidate the factors that guided physicians' treatment choices, based on the patient characteristics to be considered in hypertension management, as well as environmental characteristics that influence physicians' prescribing and adoption of new drugs [32-34]. The chart-stimulated recall interview guide was designed to start with four open-ended questions: (1) the rationale for their choice of therapy, (2) whether this was an approach used with similar patients and why, (3) how long they used this approach to treatment, and the factors that led them to adopt this strategy, and 4) the reasons for differences between the approach to the two patients (when relevant). In addition, the interview guide included a series of open-ended questions about the physician's general approach to hypertension, including: the length of time they have been treating patients for hypertension, whether they have changed their approach to hypertension treatment and why, the circumstances in which they would add a drug vs. increase the dose for existing therapy to control hypertension, the circumstances in which they would select diuretics over other classes of therapy, the sources of information used to keep informed about hypertension treatment, and the effect of the results of the ALLHAT [11] trial on their hypertension treatment choices.

Prior to each interview, the physician was informed as to which patients had been randomly selected from his/her practice so that the charts could be retrieved for consultation during the interview process. A trained interviewer was provided with the names of the two selected patients and the date and type of antihypertensive therapy that had been initially prescribed. All interviews were tape-recorded with the permission of the physicians and transcribed verbatim. The study was approved by the institutional ethics review board at McGill University. Informed consent was obtained prior to any chart stimulated recall interview.

### Analysis

Descriptive statistics were used to summarize physicians' characteristics (e.g., sex, years of practice experience) and those of their practice (i.e., mean annual patient volume, mean number of incident patients treated for uncomplicated hypertension, and mean number of patients with uncomplicated hypertension who were started on diuretics). To describe the characteristics of physicians' decision-making process regarding hypertension treatment choices, a systematic process of inductive data analysis involving content analysis was used [35-37]. Specifically, through iterative readings of the interview transcripts, a list of codes was inductively developed to characterize sentences, paragraphs or sections of the transcripts. Codes were then compared and contrasted, and similar codes were grouped into categories to delineate emerging themes that corresponded to factors characterizing physicians' decision-making process regarding hypertension treatment choices. Quotations representing these themes were extracted from the interview transcripts, and identifying information was masked to protect the confidentiality of the informants and that of their patients. The analyses were performed independently by two of the authors (CMR and JM) who then met to discuss their codification and categorization of the data. Any discrepancies were resolved through discussion among the research team.

## Results

On average, physicians who predominantly prescribe diuretics had fewer years of practice experience, saw fewer patients, and treated fewer new patients with uncomplicated hypertension (Table 1). On average, these physicians started four times as many patients with uncomplicated hypertension on a diuretic compared to physicians who tended to use other classes of treatment (28.2% vs. 7.2%) (Table 1). Three themes emerged from the analysis of the interview data. These themes differentiated physicians who predominantly prescribe diuretics as a first-line treatment of mild to moderate uncomplicated hypertension from those who predominantly prescribe drug classes other than diuretics: a) perception about the efficacy of diuretics, b) preferred approach to hypertension management, and c) perceptions about hypertension guidelines.

**Table 1:** Characteristics of the 29 physicians who participated in the study

Characteristics	Physicians who predominantly prescribe diuretics (n = 13)	Physicians who predominantly prescribe other drug classes (n = 16)
Sex	N (%)	N (%)
Female	6 (46.1)	8 (50.0)
Male	7 (53.9)	8 (50.0)
	Mean ± SD	Mean ± SD
Years of practice experience	21.9 ± 13.3	24.0 ± 5.4
Number of patients seen in 2009	1, 000.5 ± 544.3	1, 420.2 ± 743.6
Number of incident patients with uncomplicated hypertension (2009)	71.8 ± 62.9	99.3 ± 75.1
Mean proportion (%) of incident patients with uncomplicated hypertension started on diuretics (2009)	28.2% ± 11.3%	7.2% ± 4.3%

### Perceptions about the efficacy of diuretics

Physicians from both groups agreed that for patients with complicated hypertension (i.e., patients with diabetes, renal or cardiac disease) they would initiate an antihypertensive therapy with an angiotensin converting enzyme inhibitor (ACEI) or an angiotensin II receptor blocker (ARB) in order to provide these patients with the required cardiovascular or renal protection. Physicians also agreed that the target blood pressures should be < 130/80 mmHg in patients with diabetes or chronic kidney disease, and < 140/80 mmHg in patients with uncomplicated hypertension. However, physicians differed in their perceptions of the efficacy of diuretics for managing patients with mild to moderate uncomplicated hypertension. Indeed, physicians who predominantly prescribe diuretics described these drugs as being effective and as having few side effects:

"I find that diuretics are safe, effective, and inexpensive. I don't think there are any better drugs. I use them often." (MD 18)

*"Unless there is a compelling indication, I usually start with that *[i.e. diuretics]*. Even before the ALLHAT study, I was kind of doing that because I didn't think there was any benefit to one over the other, but with ALLHAT, it made it easier." *(MD 19)

"*For me a diuretic is the first line, unless there's an indication to use a different drug, such as with a diabetic patient, or a contra-indication to use diuretics, such as in a patient with a history of gout*." (MD 28)

However, among physicians who predominantly prescribe drug classes other than diuretics, the perception of diuretics was the opposite:

"We always said that diuretics are among the drugs that can cause more side effects. Then, when ACE inhibitors and angiotensin II receptor blockers and those things came along, we had far fewer side effects. And most of the time, you can't control blood pressure with a diuretic alone; diuretics for us, at least for me and for lots of people here, are mainly for second line. Even for mild hypertension, we rarely start with a diuretic." (MD 8)

"Diuretics are among those drugs that have a threshold effect. Beyond 25 mg per day, you start having all sorts of adverse effects." (MD 2)

*"In comparison to the other classes, I am very sceptical *[about the equivalent efficacy of diuretics]*; [...] I find that some classes are more effective. Some drugs are more effective than others." (MD 12)*

*"I try to use them *[i.e., diuretics]*, but they have side effects, including hyponatremia in older people. They also offer less protection against future morbidity [...] with more side effects." (MD 14)*

Physicians who predominantly prescribe drug classes other than diuretics placed a great deal of emphasis on the frequency and the number of side effects associated with using diuretics, including electrolytic disorders (e.g., hypokalemia, hyponatremia), renal failure due to dehydration, increased urinary frequency, and orthostatic hypotension. Some of these physicians also noted that diuretics alone may not be as effective as other drug classes in reaching patients' blood pressure targets. These physicians also believed that diuretics do not provide patients with the same renal and cardiovascular protection as the other drug classes, especially the angiotensin converting enzyme inhibitors (ACEIs) and the angiotensin II receptor blockers (ARBs). This last element is further illustrated by the quotations below.

"Even if you lower a patient's blood pressure with a diuretic, that doesn't mean that this drug will save his or her life later. It does not prevent infarction or what-have-you... We're not just trying to lower the numbers; we want to stop the pathology and morbidity associated with hypertension." (MD 13)

*"Diuretics may lower the blood pressure, but they don't necessarily have this protective effect on target organs that the other drug classes have" *(MD 16).

### Preferred approach to hypertension management

The physicians also had divergent opinions regarding what constitutes the "best approach to hypertension management." Physicians who predominantly prescribe diuretics described themselves as having a more gradual or weighted approach for managing patients with mild to moderate hypertension. They usually initiate antihypertensive treatments gradually. For example, they will first start the patient on a low dose of a diuretic, and gradually increase the dose as needed. Then, if the hypertension is not brought under control, they will add a low dose of a second drug class, and then optimize the dose of this drug based on the patient's response. This approach is meant to avoid the side effects that could potentially result from increasing the first drug to its maximum dose:

*"I usually increase the dose first, until they're at sort of a middling dose, like not the maximum, but the middle dose, and then, if that doesn't work out, I'll add a new one... because it s been shown that it's more efficient to add a second medication rather than increasing the dose." *(MD 28)

"*To reach my targets, I prefer to add small doses of other molecules instead of increasing the dosage of a single drug to its maximum. This is to avoid the problems of side effects*...." (MD 17)

In addition, physicians who predominantly prescribe diuretics are more cautious about using new drugs with their patients as soon as these products come out on the market. They tend to wait for the evidence about the effectiveness of the new products to accumulate before inserting these drugs into their practice:

*"I haven't used Aliskiren *[one of the newest antihypertensive] *because at the conference I went to at the University of X, they told us it is far too new to know the long-term effects." (MD 7)*

In comparison, physicians who predominantly prescribe drug classes other than diuretics as first-line treatment for mild to moderate hypertension are characterized by a more aggressive approach to treating hypertension. These physicians are notably motivated by a desire to achieve their treatment targets rapidly. They prefer to initiate an antihypertensive therapy with a drug class that they believe is more potent than diuretics, and in most cases this will be an angiotensin converting enzyme inhibitor (ACEI) or angiotensin II receptor blocker (ARB). The following quotations summarize this perspective:

"I generally follow the guidelines but not for always starting with diuretics alone; for me, at this point, it s not enough, and I don't want to waste my time." (MD 3)

"Maybe I'm wrong, but I don't think that a diuretic alone can decrease the blood pressure quite significantly. For this reason, I always start with a drug that I know will quickly and significantly decrease the blood pressure." (MD 5)

"It's not typical of my practice to prescribe diuretics alone. It's possible that they are effective in very mild hypertension but generally, for moderate hypertension, diuretics alone won t work. This is why I usually start with an ARB, very often in combination with a diuretic." (MD 16)

"For moderate hypertension, that is when the patient has a diastolic pressure of about 150 mmHg, then, I know that a thiazide diuretic will not work. For me, at this point, I will start with another drug class." (MD 20)

For these reasons, these physicians usually initiate a treatment with an ACEI or an ARB. If this treatment is not effective, they prefer to increase the dose of the selected antihypertensive to the maximum tolerated by the patient before adding another drug. This is because they believe that: 1) drug combinations are associated with greater chances of experiencing adverse effects; 2) taking more than one medication for hypertension is associated with a reduced compliance with hypertension treatment. As such, whenever they need to prescribe more than one antihypertensive drug to a patient, they will usually prefer the combination products (e.g. an ARB or an ACE combined with a thiazide diuretic). The following quotations summarize these points:

"I treat hypertension aggressively... we've always treated hypertension aggressively. If there's no side effect, I tend to increase the drug until I get to a full dose for the drug, before I add a second one." (MD 1)

*"... I think it is important to go to the maximum dose of a given drug. Because the more you add molecules, the more you increase the chances of having side effects and drug interactions." *(MD 8)

"*A lot of my patients are taking diuretics as an add-on, usually to an ACE or an ARB. Sometimes I combine a diuretic if it comes in the same pill because in terms of compliance, if the patient takes one pill, the compliance will be better than with two. So diuretics for me are mainly an add-on to and ACE or an ARB in the greatest majority of my patients... Considered that in order to give renal protection, I prefer to give them an ACE or an ARB first. And then to bring up the target I would add after a while of taking and ACE or an ARB, I would add a diuretic, usually in combination. I would try to find a drug that has a combination product." *(MD 9)

*"Well, usually I like to increase to a reasonable amount, so I'll increase until there are side effects, or to the recommended dosages, and then often for medications there's a maximum dose, but it's not usually effective over a dose that's not the maximum dose. So I'll usually go to the "maximum effective dose", not necessarily that maximum tolerated dose. And then, we'll add." *(MD 25)

Finally, physicians who predominantly prescribe drug classes other than diuretics as first-line treatment for mild to moderate hypertension are more inclined than physicians who predominantly use diuretics to prescribe new drugs as soon as they are on the market. These physicians also appear to be more influenced by the marketing activities of pharmaceutical companies or by their interactions with pharmaceutical representatives:

*"The pharmaceutical representative brought me all sorts of data about Aliskiren *[one of the newest antihypertensive]*, its efficacy, an excellent renal protection, etc. I've used it a few times already and I've found a very, very good response with no side effects; it has a kind of power, [...] it is a very interesting drug." (MD 29)*

"I have always said to myself 'it's best to take a medication that does the job well and is not expensive' but we are so 'bombarded' with encouragement to always prescribe the 'Cadillac' drug that it's hard to resist." (MD 2)

"*I think we are very solicited by pharmaceutical companies, and that they do a good job of flogging their product, since we may be prescribing too much high-performance, do-it-all drugs. I didn't remember this *[the results of the ALLHAT study regarding the efficacy of diuretics]*." *(MD 16).

### Perceptions about hypertension guidelines

Physicians from both groups agreed with evidence-based guidelines suggesting that patients with complicated hypertension (i.e., patients with diabetes, renal or cardiac disease) should be started on an angiotensin converting enzyme inhibitor (ACEI) or an angiotensin II receptor blocker (ARB) to ensure the required cardiovascular or renal protection. In addition, physicians from both groups agreed with the treatment targets put forward by these guidelines. However, physicians who predominantly prescribe drug classes other than diuretics believed that the guidelines (or the studies underlying them) put too much emphasis on the affordability of the diuretics, at the expense of their adverse effects and related compliance issues. When talking about their perceptions of the hypertension guidelines, these physicians also reiterated their doubts about the equivalent efficacy of the diuretics for lowering blood pressure:

"I think the results of the ALLHAT study are probably right... but I don't think this study assessed the side effects and the compliance issues associated with taking diuretics. It is true that diuretics are very, very cheap. But a lot of my patients stopped taking diuretics after a few days of treatment because they were constantly urinating..." (MD 4)

"My preferred first line drugs are ACEIs because I get a better control of the blood pressure with these drugs, and with fewer side effects. For me, diuretics are a second line option. So, I don t agree with the guidelines that suggest starting with diuretics, mainly due to the side effects of these drugs." (MD 11)

*"When I use diuretics in my practice, I can observe their effects on the patients. I can see that, despite what the guidelines recommend and what the ALLHAT study says, they are not that potent. When I introduce a diuretic alone versus when I use it in a combination with another drug, I can see that diuretics alone don't have a very strong impact on reducing the blood pressure" *(MD 20).

## Discussion

Hypertension is a prevalent condition worldwide, and one of the most modifiable risk factors for cardiovascular morbidity and mortality [1-5]. Despite extensive evidence suggesting that thiazide diuretics are the most cost-effective option for treating mild to moderate uncomplicated hypertension [12-16], physicians often opt for more expensive drugs [6,19]. This study sought to describe the characteristics of physicians' decision-making process regarding hypertension treatment choices.

We found that physicians who predominantly prescribe drug classes other than diuretics believe that diuretics are less effective than other drug classes in reaching patients' blood pressure targets. In addition, these physicians believe that diuretics generally have more side effects than more recent antihypertensive agents, such as angiotensin II receptor blockers (ARBs) or angiotensin converting enzyme inhibitors (ACEIs). Lastly, they believe that diuretics offer less protection against future morbidity and mortality. Of interest, there is no evidence to support these perceptions in the literature.

Indeed, systematic reviews and meta-analysis of major hypertension trials have provided evidence that low-dose diuretics are: a) as effective at lowering blood pressure as newer and more expensive agents such as ACEIs and ARBs, b) the most effective first-line treatment for preventing the occurrence of future cardiovascular morbidity and mortality, with results consistent across age and sex strata and, c) equally or better tolerated than the newer antihypertensive agents [12,16,38,39]. Despite this evidence, misperceptions about the efficacy, safety and tolerability of diuretics remain, and these misperceptions have been reported as factors that contribute to physicians' unwillingness to use diuretics for the treatment of patients with mild to moderate uncomplicated hypertension [40-42].

One potential explanation for these misperceptions is the influence of the marketing strategies employed by the pharmaceutical industry. We have indeed noted that physicians who predominantly prescribe drug classes other than diuretics appear to be more influenced by the marketing activities of pharmaceutical companies or by their interactions with pharmaceutical representatives. This observation is consistent with a growing body of literature suggesting that physicians' misperceptions about the efficacy of diuretics may result from: a) the extensive promotion of the newer drug classes at medical meetings and in medical journals and, b) the widespread overemphasis on the possible but unproven problems resulting from using diuretics [42,43]. Efforts to increase physicians' prescribing of diuretics may need to be directed at overcoming these misperceptions. In addition, an interesting area for further investigation would be an exploration of the reasons why physicians who predominantly prescribe diuretics appear to be immune to the influence of the marketing strategies employed by the pharmaceutical industry.

We also noted that physicians who predominantly prescribe drug classes other than diuretics prefer to use them as an add-on to an ACEI or an ARB in the initial phases of treatment for mild to moderate hypertension. This would appear to be contradictory. Indeed, if these physicians truly believe that diuretics cannot be used as first-line therapy for the treatment of mild to moderate hypertension due to their poorer side effects profile and their lower effectiveness in reducing patients' blood pressure compared to other drugs, why is it that diuretics can be seen as an acceptable second-line therapy? A similar inconsistency was also observed among a group of general practitioners in Germany in a recent study conducted by Kuehlein et al. [44]. It was observed that among patients who received an antihypertensive monotherapy (30.6%), only 8.6% where prescribed some diuretics and 1.5% received hydrochlorothiazide (HCT). However, among patients who received an antihypertensive combination therapy (69.4%), 79.0% received some diuretics, of which 80.8% had a combination with HCT [44]. As for Kuehlein et al. [44], we could not find any explanation as to why diuretics' side-effects perceivable by patients when used in monotherapy should disappear in combination therapy.

In addition, we found that physicians who predominantly prescribe drug classes other than diuretics are characterized by a more aggressive approach to hypertension treatment and by a desire to reach their blood pressure targets rapidly. This approach is probably desirable given that therapeutic inertia has been identified as one of the most important modifiable factors that contribute to poor blood pressure control [45]. Indeed, it is estimated that for middle-aged patients with uncomplicated hypertension, 43% of primary care physicians would not initiate pharmacological therapy unless systolic blood pressure is greater than 160 mmHg, and 33% would not initiate therapy unless diastolic blood pressure is greater than 95 mmHg [43]. Moreover, in patients without complications who are currently receiving an antihypertensive therapy, 33% of physicians would not intensify therapy for a persistent systolic blood pressure of 158 mmHg and 25% would not for a persistent diastolic blood pressure of 94 mmHg [43]. As such, the fact that physicians who predominantly prescribe drug classes other than diuretics describe themselves as being more aggressive in managing hypertension, and as wanting to achieve their blood pressure targets rapidly, is probably desirable and suggests that these physicians may be immune to clinical inertia. Additional studies are required to validate this observation, and to determine if blood pressure control rates differ between physicians who predominantly prescribe diuretics and those who predominantly prescribe other drug classes. While having an aggressive approach to the management of hypertension may be desirable, the strategy used by physicians who predominantly prescribe drug classes other than diuretics for reaching their blood pressure target may be questioned. Indeed, we have noted that these physicians often prefer to increase the dose of their initial treatment choice--which is often an ACEI or an ARB--to the maximum tolerated by the patient before adding a medication from another drug class. However, studies have shown that single drug therapy, even when maximally titrated, is at best only modestly effective in normalizing blood pressure for patients with mild to moderate hypertension [46]. Indeed, in most patients for which blood pressure targets are difficult to achieve the use of multidrug combinations will often produce greater blood pressure reduction at lower doses of the component agents, resulting in fewer adverse events [47,48].

An important strength of our study was the use of the chart stimulated recall interview approach. This method allowed us to interview physicians about their actual practice decisions with real hypertensive patients. This method offers new insights over existing methods, such as manual chart review, surveys or hypothetical case studies, as it allows physicians to reflect on their current prescribing behaviors for patients in their practice and to comment on the rationale for their treatment selection. In addition, with the growing availability of electronic medical records, the approach used in this study offers new and very interesting avenues for qualitative researchers interested in studying physicians' performance.

Our study also has several limitations. First, it was limited to a jurisdiction where a significant proportion of the vulnerable population is covered by a public drug plan. Factors influencing physicians' decisions may thus differ in other jurisdictions where patients are paying for their drugs. In addition, we investigated the factors influencing physicians' treatment choices for a single health condition. It is possible that these factors vary by type of health problem. Therefore, additional studies conducted with patients in other jurisdictions and with patients presenting a variety of health conditions are recommended.

## Conclusions

Thiazide diuretics are cost-effective for the treatment of mild to moderate uncomplicated hypertension, but physicians often opt for more expensive treatment options such as angiotensin II receptor blockers (ARBs) or angiotensin converting enzyme inhibitors (ACEIs). This study sought to describe the characteristics of physicians' decision-making process regarding hypertension treatment choices. We found that physicians who predominantly prescribe drug classes other than diuretics appear to have several misperceptions regarding the efficacy, the safety and the tolerability of diuretics. Our data also indicate that the marketing strategies employed by the pharmaceutical industry may contribute to these misperceptions. Efforts to increase physicians' prescribing of diuretics may need to be directed to overcoming misunderstandings about the effectiveness and tolerability of these medicines.

## Competing interests

The authors declare that they have no competing interests.

## Authors' contributions

All listed authors (CMR, JM, RMT): have made substantial contributions to conception and design, or acquisition of data, or analysis and interpretation of data; have been involved in drafting the manuscript or revising it critically for important intellectual content; and have given final approval of the version to be published. All authors read and approved the final manuscript.

## Pre-publication history

The pre-publication history for this paper can be accessed here:

http://www.biomedcentral.com/1471-2296/13/9/prepub
